# “Age matters”—German claims data indicate disparities in lung cancer care between elderly and young patients

**DOI:** 10.1371/journal.pone.0217434

**Published:** 2019-06-12

**Authors:** Julia Walter, Amanda Tufman, Rolf Holle, Larissa Schwarzkopf

**Affiliations:** 1 German Research Center for Environmental Health, Institute for Health Economics and Health Care Management (IGM), Helmholtz Zentrum München, Germany; 2 German Center for Lung Research (DZL), Gießen, Germany; 3 Ludwig-Maximilians-University Hospital (LMU) Munich, Medical Clinic V–Pneumology, Munich, Germany; University of South Alabama Mitchell Cancer Institute, UNITED STATES

## Abstract

**Background:**

Although lung cancer is most commonly diagnosed in elderly patients, evidence about tumor-directed therapy in elderly patients is sparse, and it is unclear to what extent this affects treatment and care. Our study aimed to discover potential disparities in care between elderly patients and those under 65 years of age.

**Methods:**

We studied claims from 13 283 German patients diagnosed with lung cancer in 2009 who survived for at least 90 days after diagnosis. We classified patients as “non-elderly” (≤ 65), “young-old” (65–74), “middle-old” (75–84), and “old-old” (≥ 85). We compared receipt of tumor-directed therapy (6 months after diagnosis), palliative care, opioids, antidepressants, and pathologic diagnosis confirmation via logistic regression. We used generalized linear regression (gamma distribution) to compare group-specific costs of care for 3 months after diagnosis. We adjusted all models by age, nursing home residency, nursing care need, comorbidity burden, and area of residence (urban, rural). The age group “non-elderly” served as reference group.

**Results:**

Compared with the reference group “non-elderly”, the likelihood of receiving any tumor-directed treatment was significantly lower in all age groups with a decreasing gradient with advancing age. Elderly lung cancer patients received significantly fewer resections and radiotherapy than non-elderly patients. In particular, treatment with antineoplastic therapy declined with increasing age (“young-old” (OR = 0.76, CI = [0.70,0.83]), “middle-old” (OR = 0.45, CI = [0.36,0.50]), and “old-old” (OR = 0.13, CI = [0.10,0.17])). Patients in all age groups were less likely to receive structured palliative care than “non-elderly” (“young-old” (OR = 0.84, CI = [0.76,0.92]), “middle-old” (OR = 0.71, CI = [0.63,0.79]), and “old-old” (OR = 0.57, CI = [0.44,0.73])). Moreover, increased age was significantly associated with reduced quotas for outpatient treatment with opioids and antidepressants. Costs of care decreased significantly with increasing age.

**Conclusion:**

This study suggests the existence of age-dependent care disparities in lung cancer patients, where elderly patients are at risk of potential undertreatment. To support equal access to care, adjustments to public health policies seem to be urgently required.

## Introduction

Lung cancer was the fourth leading cause of death in Germany in 2016 [1]. Among all types of cancer, lung cancer accounted for the highest proportion of cancer-related deaths in men and the second highest in women [1]. Lung cancer is most commonly diagnosed in elderly patients with median age at diagnosis of around 68–70 years in developed countries [2–4]. In Germany in 2013, incidence rates ranged from 125 per 100 000 in 55- to 59-year-old men to 423 in 100 000 in 80- to 84-year-old men, and from 64 to 106 per 100 000 in women [5]. Despite these numbers, historically, elderly patients are underrepresented in clinical trials [6]; therefore, evidence on treatment effects in this relevant patient group is insufficient. Efforts to address this issue by enrolling more elderly patients in clinical trials have been made [7] and, since the 1990s, numbers have improved [8]. However, most elderly trial participants are enrolled in age-unspecific trials [9]. In these trials, only patients who meet the strict eligibility criteria concerning comorbidities and performance status are included [6, 9, 10]; therefore, they most probably do not represent the average elderly patient [10]. Recent guidelines include recommendations for elderly patients (>70 years), for example relating to performance status, but do not further differentiate between subgroups of elderly patients, for instance young-old, middle-old, and old-old [11]. However, clinical experience suggests that differences exist between age-based subgroups of elderly patients, and that both chronological and biological age can be relevant to treatment decisions in the setting of lung cancer [12]. Numerous studies have shown that treatments effective in younger adults can be of similar benefit to elderly patients [13, 14]; however, there is also evidence that treatment approaches in elderly patients are considerably different [15, 16]. Furthermore, survival in lung cancer is significantly associated with age as well [17, 18]. A study from Turkey found that patients over the age of 70 years had 1-year survival rates of 42.5% compared with 67.3% in patients 70 years or younger [17]. Similarly, in an analysis of Surveillance, Epidemiology and End Results data, an age gradient of 7.4% vs. 12.3% vs. 15.5% was found concerning 5-year survival rates in lung cancer patients aged younger than 70 years, between 70 und 79 years, and 80 years or older respectively [18].

Although most of the trials and studies concerning the treatment of elderly lung cancer patients focus on tumor-directed therapy, and some include pain management, no study has yet looked at differences in therapy comprehensively. Although access to tumor-directed treatment is certainly of importance, access to diagnostic tests and palliative care is also crucial and should be available to all affected patients regardless of age. Too little is known about care provided to elderly patients in various age subgroups.

Therefore, this study comparing non-elderly and elderly lung cancer patients in Germany aimed to:

1. evaluate whether tumor-directed treatment differs depending on age,
2. investigate whether delivery of diagnostic and palliative care measures is different across age groups, and
3. compare the costs of initial treatment and care after diagnosis across three elderly age groups.

## Materials and methods

### Dataset and sample selection

We analyzed anonymized health insurance claims from 17 478 patients with a first diagnosis of lung cancer in 2009—identified via ICD10 code C34—provided by the Scientific Institute of the AOK health insurance trust (WIdO), covering about 30% of the German population. As our dataset contained only retrospectively achieved and anonymized information on patients, meaning that the person in charge of this examination cannot make any inference to the individual person’s data, the Ethics Committee of the Medical Faculty of the Ludwig-Maximilians-University of Munich approved exhaustive analyses of the data (Votum-Number 88–15). Additionally, consultation of an ethics committee is generally not required for this kind of study [19]. Basic data contained birth date, sex, postal codes, care level, and nursing home residency status over the course of the disease. Care level indicates the intensity of assistance needed to complete activities of daily living (higher care level = more assistance) [20]. Additionally, we used claims for hospitalization, outpatient hospital care, outpatient doctor visits, and medications including German International Classification of Diseases (ICD-10-GM) codes, OPS codes (German version of the International Classification of Procedures in Medicine), billing codes (GONR), and Anatomical Therapeutic Chemical (ATC) codes.

We identified 17 478 patients with incident lung cancer in 2009 according to a three-step process. First, we selected all patients with a diagnosis of lung cancer (ICD-C34) in 2009. In a second step, to avoid false positives, we included only patients with at least one inpatient principal diagnosis or at least two confirmed outpatient diagnoses in two distinct quarters of the year 2009. Third, we excluded all patients with a history of lung cancer or lung metastases in the 2 years prior to 2009. Further detailed information about this process can be obtained elsewhere [21]. From the sample of patients with incident lung cancer, we excluded patients with missing postal code of residence (n = 176). Additionally, we excluded those who died within the first 3 months after diagnosis (n = 4 019), as treatment options for them are limited and time to organize care is short. The proportion of these patients increased by age group, which we considered a source of bias, leading to an overestimation of treatment disparities. The remaining 13 283 patients were considered to be medically stable enough to qualify for some type of care.

We classified our sample according to age at time of diagnosis into “non-elderly” (≤ 65 years), “young-old” (65–74 years),” middle-old” (75–84 years), and “old-old” (≥ 85 years), a definition regularly used in gerontology [22].

Claims data do not include information about the stage of lung cancer, but the stage impacts on eligibility for therapeutic procedures [23]. Therefore, we stratified our sample into patients with advanced stage (with metastases) and patients with early stage (without metastases) disease, using the ICD codes for metastases within the 3 months after lung cancer diagnosis.

### Outcome parameters

We compared the type of tumor-directed therapy in the 6 months after diagnosis by identifying antineoplastic therapy (cytostatic chemotherapy and targeted therapy), radiotherapy, and surgical lung resections from claims in the hospital (ICD and OPS codes), outpatient doctor visits (ICD and GONR codes), and prescribed medications (ATC codes). For information on specific codes, please refer to Schwarzkopf et al. [21].

In patients not receiving any tumor-directed treatments, we analyzed whether they received further invasive diagnostic measures (biopsy) to confirm the histological diagnosis.

To measure palliative care treatments, we first compared the proportion of patients who had claims for either inpatient (OPS codes) or outpatient (GONR codes) multimodal, structured palliative care, among patients who were deceased during the observation period. Second, we calculated the number of days between the first diagnosis and the first structured palliative care treatment for patients receiving structured palliative care. Third, as pain management and treatment of psychosocial aspects are an integral part of palliative care, we assessed the proportion of patients receiving opioids or antidepressants through claims for outpatient medications (ATC codes).

Additionally, we assessed all-cause and lung cancer-specific total, inpatient, outpatient, and medication expenditures within the 3 months after diagnosis to reflect the immediate treatment and care intensity. Lung cancer-specific expenditures related to inpatient visits with a primary diagnosis of lung cancer, medications used in antineoplastic therapy or as supportive drugs (e.g., antiemetics, antianemics), and outpatient cases with a diagnosis of lung cancer.

### Confounders

We included the following variables as confounders in all analyses based on strong evidence in the literature: baseline information on sex, nursing home residency, and need for nursing care (care level) as a measure of performance status, comorbidity burden (Charlson index) [24], and residential area (rural/urban) [25].

Sex may influence the biology of lung cancer and, therefore, treatment options and outcomes [26, 27]. Also, we expected that the proportions of males and females in each age group may differ. Care level in combination with nursing home residency and Charlson index reflects the patients’ frailty, which has been shown to influence treatment choice and outcomes in lung cancer specifically in elderly patients [28–30]. The Charlson index was calculated based on all inpatient ICD and all confirmed outpatient diagnoses in the 2 years prior to lung cancer diagnosis (excluding diagnoses of lung cancer and metastases). The area of residence has also been shown to influence treatments and survival in lung cancer [31, 32]. We identified the type of district of residence in our dataset based on the postal code of residence, using district types defined by the German Federal Institute for Research on Building, Urban Affairs and Spatial Development for 2014 [33].

### Statistical analysis

In the univariate analysis, we calculated care as proportions and costs as means for the four age groups. We compared proportions using Chi^2^ test and means using Kruskal–Wallis test.

For our multivariate analysis, we used logistic regression models for the binary outcomes palliative care, opioid medication, antidepressant medication, biopsy, no tumor-directed treatment, antineoplastic therapy, radiotherapy, and tumor resection, all reported as odds ratios (OR).

For count data (time until first palliative care), we used generalized linear regression models with negative-binomial distribution and reported results as incidence rate ratios (IRR). To model costs, we used a generalized linear model with gamma distribution and calculated age group-specific average costs based on recycled predictions. We applied non-parametric bootstrapping (1 000 bootstrap repetitions, percentile method) to obtain confidence intervals (CI) and p-values. Recycled predictions were obtained from the gamma regression model by averaging predicted scores, after fixing the value of one independent variable (here, age group “non-elderly”) and using observed values on the remaining independent variables (age groups “young-old”, “middle-old”, “old-old”). The recycled predictions then provide adjusted means for all age groups [34].

We used the age group “non-elderly” as reference in all regression models and adjusted them by the confounders mentioned above. We used a significance threshold of α ≤ 0.05 with 95% CI for all outcomes.

All analyses were performed using SAS 9.3 (SAS software from the SAS System for Microsoft, Version 9.3 (c) 2002–2010 by SAS Institute Inc., Cary, NC, USA); tables and figures were created in Microsoft Excel and R Studio using the graphical package *forestplot* [35].

### Sensitivity analysis

To address a bias from possible misclassification of patients to the strata of no metastases, we conducted a sensitivity analysis. A misclassification could happen if, after the initial diagnosis of lung cancer, no further diagnostic procedures to investigate the extent of the disease (presence of metastases) were performed, due to either patient refusal or other concerns. Therefore, in this sensitivity analysis, we only included patients who did not have a diagnosis of metastases, but who had received further diagnostic procedures, including but not limited to, PET-CT, abdominal or cranial CT, and MRI.

## Results

### Sample characteristics and univariate analysis

Of the 13 283 patients, 4 595 (34.6%) were classified as “non-elderly”, 5 195 (39.1%) as “young-old”, 3 107 (23.4%) as “middle-old”, and 386 (2.9%) as “old-old”. Within all four age groups, the majority of patients were male, with the highest proportion among “young-old” (74.4%) and the lowest among “old-old” (55.7%). Patients’ frailty, as reflected in comorbidity burden, nursing home status, and care level, increased by age group. In all age groups, most patients lived in urban areas, with the highest proportion of urban dwellers among “non-elderly”. Around 60% of all patients had a diagnosis of metastases in the 3 months after diagnosis. In these patients, the proportion of “non-elderly” patients was around 10% points higher compared with patients without metastases. In consequence, in patients without metastases, a higher proportion of patients was in the age groups “middle-old” and “old-old” than in the stratum of patients with metastases. Further characteristics of patients with metastases compared with those without metastases did not differ. Table 1 displays sample characteristics of the whole study population, as well as for patients with and without metastases.

**Table 1:** Sample characteristics of lung cancer patients diagnosed in 2009 in Germany.

All patients					
Means and proportions	Young (n = 4 595)	Young-old (n = 5 195)	Middle-old (n = 3 107)	Old-old (n = 386)	p-value
Male (%)	66.5	74.4	68.9	55.7	< 0.0001
Mean Charlson index at diagnosis	1.7	2.7	3.2	3.1	< 0.0001
Nursing home residency (%)	1.1	1.8	2.6	12.4	< 0.0001
*Care level (%)*					
No care level	91.7	86.8	77.2	53.9	< 0.0001
Care level 1	5.1	8.1	14.9	26.4	
Care level 2	2.7	4.4	6.9	16.8	
Care level 3	0.5	0.8	1.0	2.9	
Urban residence area (%)	66.4	61.8	60.6	60.1	< 0.0001
Patients with metastases					
Means and proportions	Young (n = 1 616)	Young-old (n = 2 077)	Middle-old (n = 1 492)	Old-old (n = 225)	p-value
Male (%)	65.0	75.6	68.8	55.1	<0.0001
Mean Charlson index at diagnosis	1.7	2.9	3.3	3.3	< 0.0001
Nursing home residency (%)	1.3	1.5	3.2	13.3	<0.0001
*Care level (%)*					
No care level	93.4	88.9	77.6	52.4	<0.0001
Care level 1	4.3	7.1	14.8	26.7	
Care level 2	1.9	3.5	6.6	18.7	
Care level 3	0.4	0.5	1.0	2.2	
Urban residence area (%)	66.7	62.5	60.5	58.7	0.001
Patients without metastases					
Means and proportions	Young (n = 2 979)	Young-old (n = 3 118)	Middle-old (n = 1 615)	Old-old (n = 161)	p-value
Male (%)	67.2	73.7	69.0	56.5	<0.0001
Mean Charlson index at diagnosis	1.7	2.6	3.1	2.9	<0.0001
Nursing home residency (%)	1.0	1.9	2.1	11.2	<0.0001
*Care level (%)*					
No care level	90.8	85.3	76.8	55.9	<0.0001
Care level 1	5.4	8.7	15.0	26.1	
Care level 2	3.2	4.9	7.2	14.3	
Care level 3	0.6	1.0	1.0	3.7	
Urban residence area (%)	66.3	61.4	60.7	62.1	0.0001

Notes: Means and proportions of sample characteristics in all patients and both metastases strata, in age groups “non-elderly”(≤ 65 years),” young-old” (65–74 years), “middle-old” (75–84 years), and “old-old” (≥ 85 years). P-values from Chi^2^ test for binary variables and Kruskal–Wallis test for continuous variables.

Within the pooled cohort of patients with and without metastases, proportions and means of all unadjusted outcomes differed significantly between the age groups, with higher age indicating lower treatment intensity. Additionally, domain-specific unadjusted expenditures differed significantly between the distinct age groups, with highest expenditures in “non-elderly” and gradually decreasing expenditures among more advanced age groups.

In patients without metastases, the proportion of patients receiving palliative care was higher than in patients with metastases across all age groups. Patients in higher age groups were less likely to receive palliative care than younger patients in both metastases strata. Prescription of opioids and antidepressants decreased with increasing age group independent of whether patients had metastases at diagnosis or not. The proportion of patients receiving opioids was higher in patients with metastases; the proportion of antidepressant prescriptions was similar. Regarding tumor-directed treatment, the share of patients receiving no tumor-directed therapy was higher in patients without metastases than in patients with metastases and increased with increasing age group. Diagnosis confirmation through biopsy was higher in patients with metastases than in patients without metastases and decreased with increasing age. Therapy with antineoplastic agents decreased with increasing age group irrespective of the presence of metastases. Although there was no clear trend of radiotherapy treatment in patients without metastases, the proportion of patients with radiotherapy treatment decreased with increasing age in patients with a metastases diagnosis at diagnosis of lung cancer. Regarding lung cancer resection, there was no significant difference related to age in patients with metastases. In patients without metastases, the share of patients with a tumor resection decreased with increasing age group.

Results of the univariate analysis in the whole sample and in both metastases strata are displayed in Table 2.

**Table 2:** Unadjusted means and proportions of care and expenditures of lung cancer patients diagnosed in 2009 in Germany.

	All patients				
	Young (n = 4 595)	Young-old (n = 5 195)	Middle-old (n = 3 107)	Old-old (n = 386)	p-value
Structured palliative care in deceased patients (%) (n)	29.8 (1 368)	27.0 (1 402)	25.6 (795)	23.8 (92)	0.0001
of these, mean time until structured palliative care (sd)	369.1 (294)	356.8 (306)	312.2 (278)	253.4 (223)	<0.0001
Opioid medication (%) (n)	70.8 (3 255)	65.3 (3 393)	63.5 (1 974)	58.8 (227)	<0.0001
Antidepressants in patients without prior diagnosis of depression (%) (n)	30.8 (1 198)	25.8 (1 178)	22.4 (605)	18.2 (55)	<0.0001
No tumor-directed treatment (%) (n)	4.4 (200)	7.8 (403)	20.2 (626)	54.7 (211)	<0.0001
of these, patients with biopsy (%) (n)	61.5 (123)	60.3 (243)	56.2 (352)	43.6 (92)	0.0003
Antineoplastic therapy (%) (n)	65.9 (3 030)	58.3 (3 027)	44.3 (1 376)	17.1 (66)	<0.0001
Radiotherapy (%) (n)	23.7 (1 090)	21.9 (1 139)	21.4 (664)	17.1 (66)	0.004
Tumor resection (%) (n)	34.7 (1 595)	35.4 (1 839)	27.8 (864)	15.3 (59)	<0.0001
Mean all-cause total expenditures (€)	12 822	11 954	10 125	6 695	<0.0001
Mean all-cause hospital expenditures	10 393	9 790	8 411	5 491	<0.0001
Mean all-cause outpatient expenditures	101	906	780	670	<0.0001
Mean all-cause medication expenditures	1 418	1 258	934	534	<0.0001
Mean lung cancer-specific total expenditures	10 168	9 371	7 669	4 330	<0.0001
	No metastases				
	Young (n = 1 616)	Young-old (n = 2 077)	Middle-old (n = 1 492)	Old-old (n = 225)	p-value
Structured palliative care in deceased patients (%) (n)	19.9 (322)	19.6 (407)	19.4 (289)	26.7 (60)	0.08
of these, mean time until structured palliative care (sd)	488.0 (327)	441.1 (326)	373.8 (304)	300.3 (239)	<0.0001
Opioid medication (%) (n)	64.7 (1 045)	59.6 (1 237)	59.5 (887)	56.0 (126)	0.002
Antidepressants in patients without prior diagnosis of depression (%) (n)	31.2 (424)	25.5 (461)	20.3 (261)	18.8 (33)	<0.0001
No tumor-directed treatment (%) (n)	7.1 (114)	10.4 (215)	25.5 (381)	65.8 (148)	<0.0001
of these, patients with biopsy (%) (n)	62.3 (71)	58.6 (126)	54.9 (209)	39.9 (59)	0.001
Antineoplastic therapy (%) (n)	45.1 (728)	39.6 (823)	30.9 (461)	12.0 (27)	<0.0001
Radiotherapy (%) (n)	13.7 (221)	16.4 (341)	18.5 (276)	13.3 (30)	0.002
Tumor resection (%) (n)	54.6 (882)	50.8 (1 054)	34.3 (511)	11.6 (26)	<0.0001
Mean all-cause total expenditures (€)	11 381	10 645	9 181	5 920	<0.0001
Mean all-cause hospital expenditures	9 515	8 856	7 612	4 718	<0.0001
Mean all-cause outpatient expenditures	812	812	793	724	0.37
Mean all-cause medication expenditures	1 016	950	760	478	0.20
Mean lung cancer-specific total expenditures	9 000	8 126	6 583	3 708	<0.0001
	Metastases				
	Young (n = 2 979)	Young-old (n = 3 118)	Middle-old (n = 1 615)	Old-old (n = 161)	p-value
Structured palliative care in deceased patients (%) (n)	35.1 (1 046)	31.9 (995)	31.3 (506)	19.9 (32)	<0.0001
of these, mean time until structured palliative care (sd)	332.5 (273)	322.3 (290)	277.1 (256)	165.5 (158)	<0.0001
Opioid medication (%)(n)	74.2 (2 210)	69.2 (2 156)	67.3 (1 087)	62.7 (101)	<0.0001
Antidepressants in patients without prior diagnosis of depression (%) (n)	30.6 (774)	26 (717)	24.4 (344)	17.3 (344)	<0.0001
No tumor-directed treatment (%) (n)	2.9 (86)	6.0 (188)	15.2 (245)	39.1 (63)	<0.0001
of these, patients with biopsy (%) (n)	60.5 (52)	62.2 (117)	58.4 (143)	52.4 (33)	0.56
Antineoplastic therapy (%) (n)	77.3 (2 302)	70.7 (2 204)	56.7 (915)	24.2 (39)	<0.0001
Radiotherapy (%) (n)	29.2 (869)	25.6 (798)	24.0 (388)	22.4 (36)	0.0003
Tumor resection (%) (n)	23.9 (713)	25.2 (785)	21.9 (353)	20.5 (33)	0.06
Mean all-cause total expenditures (€)	13 604	12 826	10 997	7 778	<0.0001
Mean all-cause hospital expenditures	10 849	10 395	9 135	6 571	<0.0001
Mean all-cause outpatient expenditures	1 120	969	767	596	<0.0001
Mean all-cause medication expenditures	1 635	1 463	1 094	611	<0.0001
Mean lung cancer-specific total expenditures	10 422	9 823	8 440	5 127	<0.0001

Notes: Means and proportions of care and expenditures in age groups “non-elderly”(≤ 65 years),” young-old” (65–74 years), “middle-old” (75–84 years), and “old-old” (≥ 85 years). All-cause and lung cancer-specific total, inpatient, outpatient, and medication expenditures within the3 months after diagnosis. Lung cancer-specific expenditures relate to inpatient visits with a primary diagnosis of lung cancer, medications used in antineoplastic therapy or as supportive drugs (e.g., antiemetics, antianemics), and outpatient cases with a diagnosis of lung cancer.

P-values from Chi^2^ test for binary variables and Kruskal–Wallis test for continuous variables.

### Multivariate analysis

Results of the multivariate analysis of the whole sample can be found in S1 (care) and S2 (costs) Figs in the supplementary material.

#### Patients without metastases

**Fig 1:**
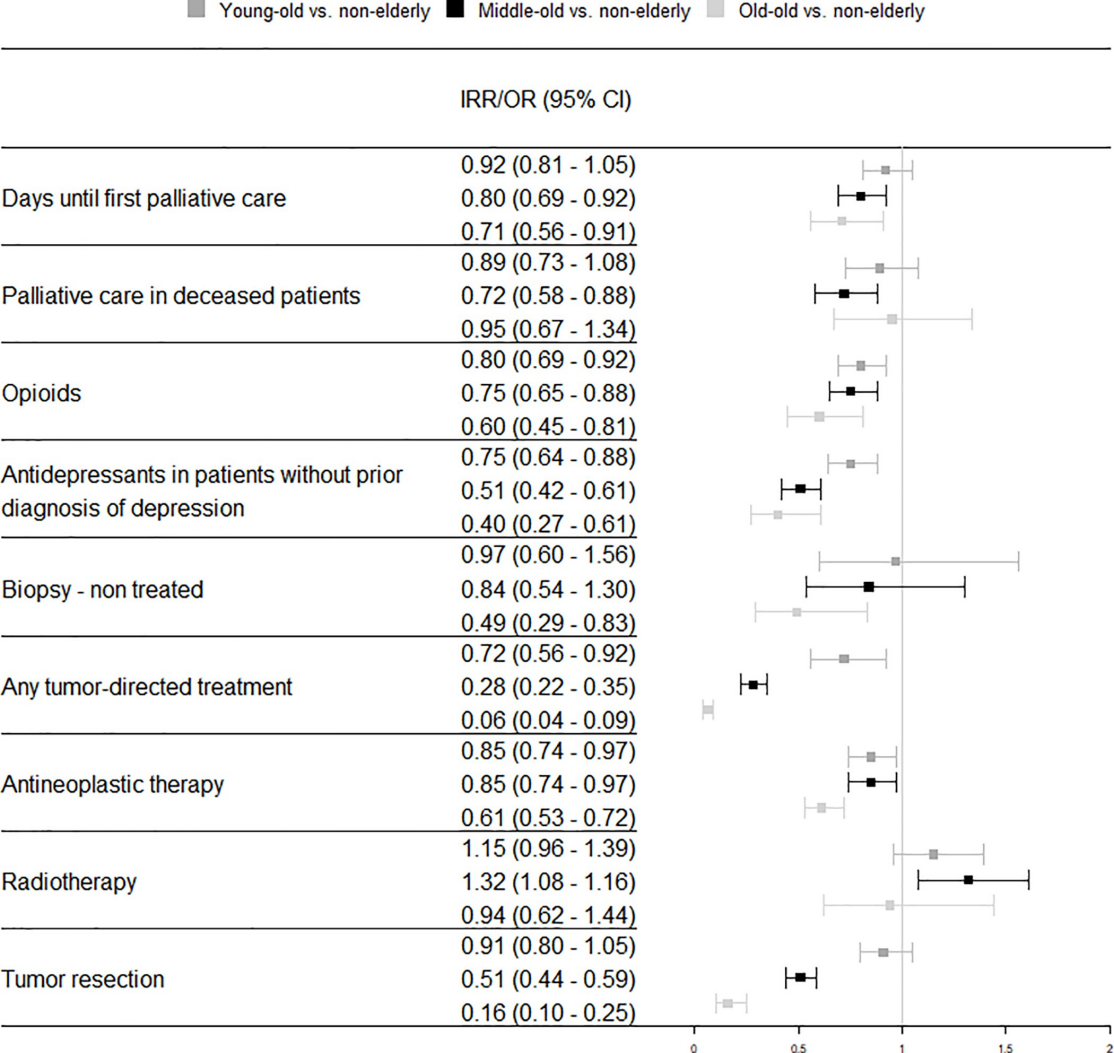
Adjusted odds ratio and incidence rate ratio of care among age groups of lung cancer patients with metastases diagnosed in 2009 in Germany. Time until first palliative care is reported as incidence rate ratio (IRR); all other outcomes are reported as odds ratios (OR). All IRR and ORs are adjusted for sex, nursing home residency, care level, Charlson comorbidity index, and rural vs. urban residence. CI = confidence interval, OR = odds ratio.

No clear trend was observed in patients without metastases concerning structured palliative care. Compared with the reference group “non-elderly”, only “middle-old” patients showed a significantly lower likelihood of receiving structured palliative care (OR = 0.72, CI = [0.58, 0.88]). Time until palliative care was significantly shorter in “non-elderly” patients compared with “middle-old” (IRR = 0.80, CI = [0.69, 0.92]) and “old-old” patients (IRR = 0.71, CI = [0.56, 0.91]). All elderly age groups had a significantly lower chance of receiving opioids and antidepressants compared with “non-elderly” patients. Compared with “non-elderly” patients, the likelihood of receiving any tumor-directed treatment was significantly lower in all age groups with a decreasing gradient with advancing age. Referring to patients with no tumor-directed treatment, diagnostic biopsies were performed significantly less often in “old-old” than in “non-elderly” patients (OR = 0.49, CI = [0.29, 0.83]). The odds of receiving antineoplastic therapy were significantly lower and decreased with increasing age group. Receiving radiotherapy was significantly more likely in “middle-old” patients than in “non-elderly” patients (OR = 1.32, CI = [1.08, 1.16]). The likelihood of tumor resection was significantly higher in “non-elderly” patients compared with “middle-old” and “old-old” patients. All results from the multivariate analysis of patients without metastases are available in Fig 1.

#### Patients with metastases

**Fig 2:**
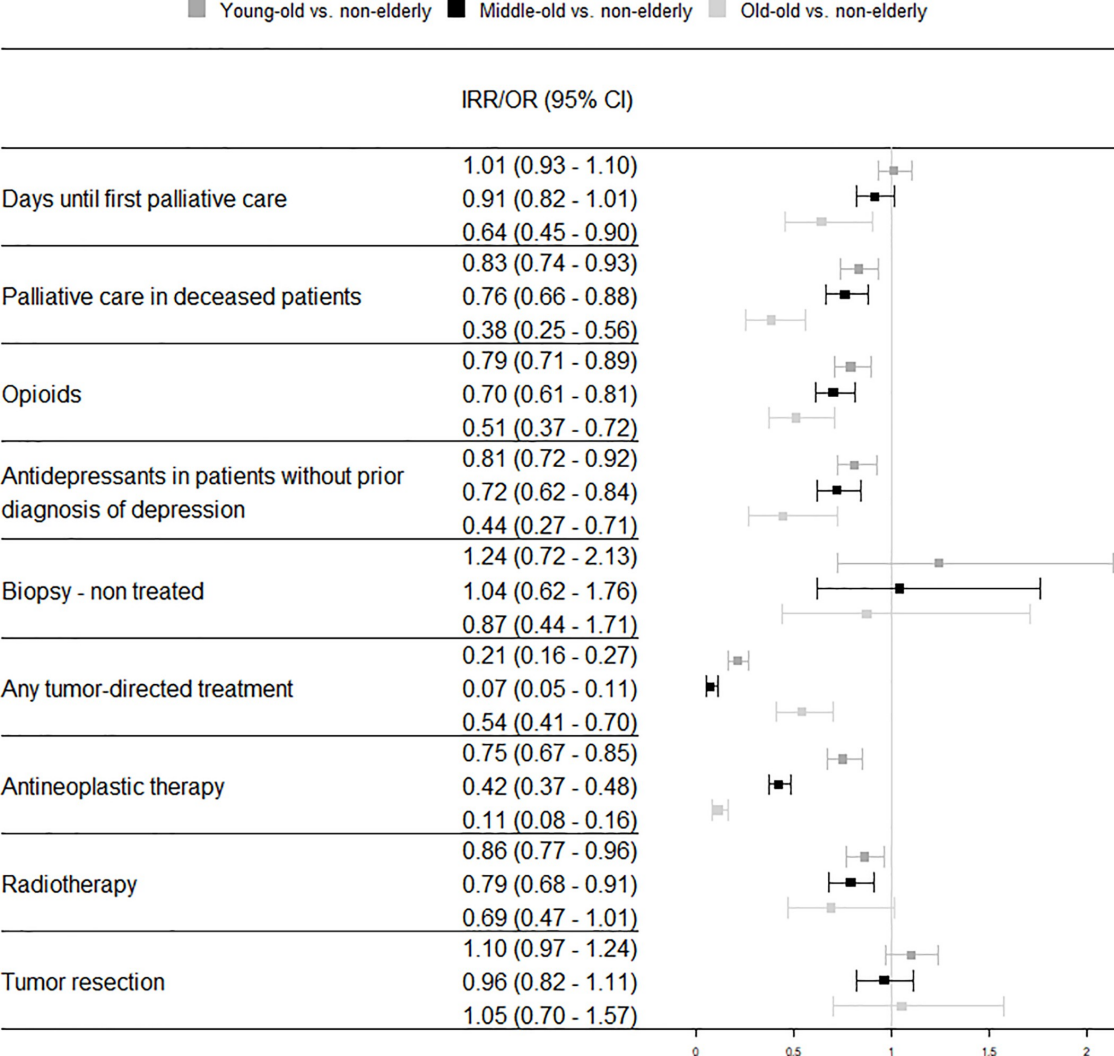
Adjusted odds ratio and incidence rate ratio of care among age groups of lung cancer patients without metastases diagnosed in 2009 in Germany. Time until first palliative care is reported as incidence rate ratio (IRR); all other outcomes are reported as odds ratios (OR). All IRR and ORs are adjusted for sex, nursing home residency, care level, Charlson comorbidity index, and rural vs. urban residence. CI = confidence interval, OR = odds ratio.

The likelihood of receiving palliative care was significantly lower in all age groups compared with “non-elderly” patients in the group of patients with metastases (“young-old”: OR = 0.83, CI = [0.74, 0.93], “middle-old”: OR = 0.76, CI = [0.66, 0.88], “old-old”: OR = 0.38, CI = [0.25, 0.56]). Similar to patients without metastases, in patients with metastases, the odds of receiving opioid and antidepressant medication was also significantly lower and decreased with increasing age group. Patients in all age groups were less likely to receive any kind of tumor-directed therapy compared with “non-elderly” patients, but the likelihood of diagnosis confirmation in untreated patients did not differ. Patients in all age groups were less likely to receive antineoplastic therapy than “non-elderly” patients (“young-old”: OR = 0.75, CI = [0.67, 0.85], “middle-old”: OR = 0.42, CI = [0.37, 0.48], “old-old”: OR = 0.11, CI = [0.08, 0.16]). Additionally, the odds of receiving radiotherapy were lower in the “middle-old” and “old-old”. The likelihood of receiving a resection was not associated with age in patients with metastases. Adjusted odds ratios and incidence rate ratios of patients with metastases are shown in Fig 2.

**Fig 3:**
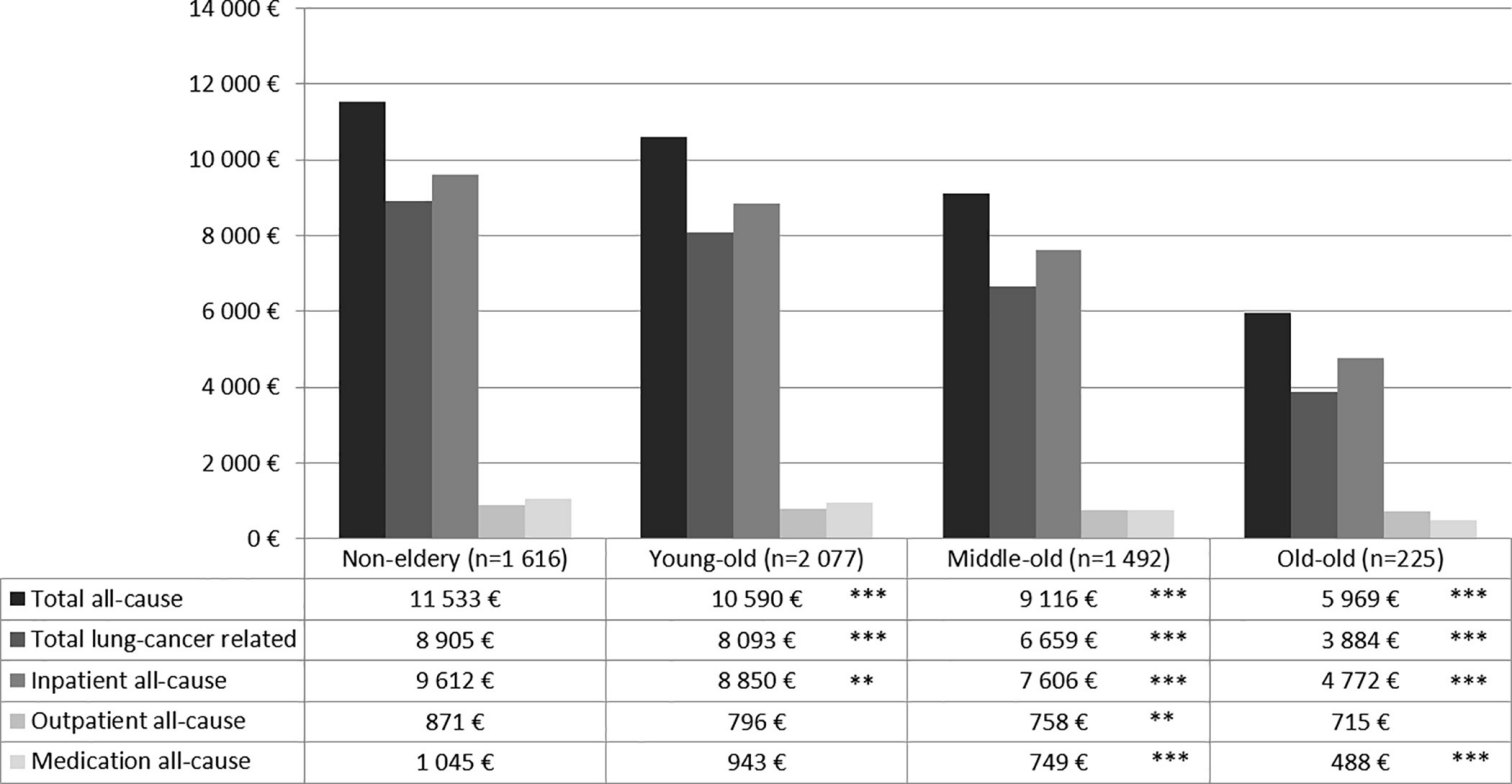
Adjusted mean expenditures in the 3 months after diagnosis among age groups in lung cancer patients with metastases diagnosed in 2009 in Germany. All-cause and lung cancer-specific total, inpatient, outpatient, and medication expenditures within the 3 months after diagnosis reported as recycled predictions with 95% confidence intervals. Significance levels (* <0.05, ** <0.01, *** < 0.0001) indicate significant differences between the age groups “young-old” (65–74 years), “middle-old” (75–84 years), and “old-old” (≥ 85 years) and the reference group “non-elderly” (≤ 65 years). Lung cancer-specific expenditures relate to inpatient visits with a primary diagnosis of lung cancer, medications used in antineoplastic therapy or as supportive drugs (e.g., antiemetics, antianemics), and outpatient cases with a diagnosis of lung cancer.

**Fig 4:**
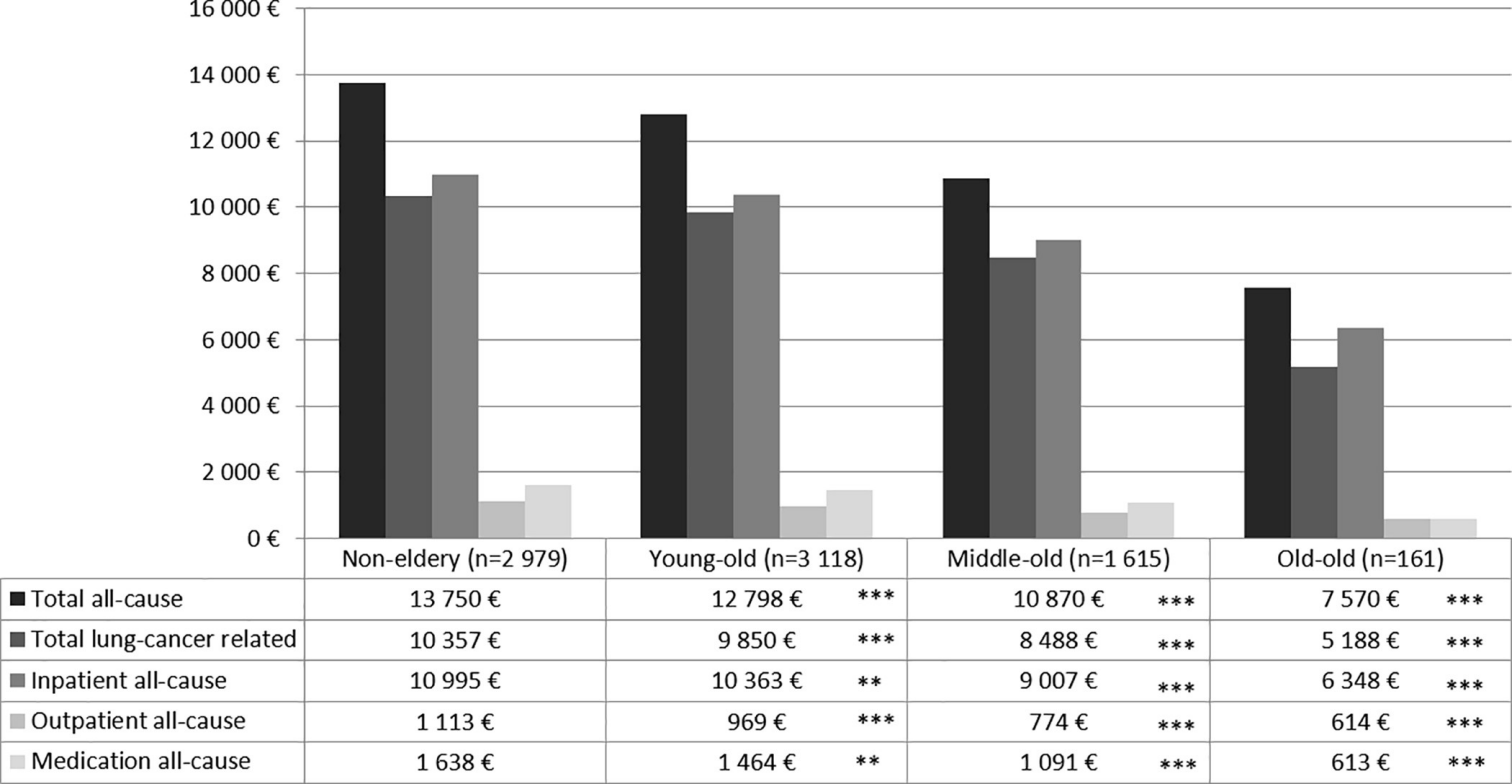
Adjusted mean expenditures in the 3 months after diagnosis among age groups in lung cancer patients without metastases diagnosed in 2009 in Germany. All-cause and lung cancer-specific total, inpatient, outpatient, and medication expenditures within the 3 months after diagnosis reported as recycled predictions with 95% confidence intervals. Significance levels (* <0.05, ** <0.01, *** < 0.0001) indicate significant differences between the age groups “young-old” (65–74 years), “middle-old” (75–84 years), and “old-old” (≥ 85 years)and the reference group “non-elderly” (≤ 65 years). Lung cancer-specific expenditures relate to inpatient visits with a primary diagnosis of lung cancer, medications used in antineoplastic therapy or as supportive drugs (e.g., antiemetics, antianemics), and outpatient cases with a diagnosis of lung cancer.

Figs 3 and 4 show age group-specific expenditures as recycled predictions with CIs. All types of expenditures decreased by age group, with “non-elderly” patients incurring significantly higher expenditures in all domains than the three more advanced age groups in patients with metastases. In patients without metastases, outpatient all-cause expenditures did not differ significantly between “non-elderly” and “young-old” as well as “old-old” patients. Additionally, all-cause medication expenditures did not differ between “non-elderly” and “old-old” patients. Other than that, the results were the same as in patients without metastases.

### Sensitivity analysis

Results of the sensitivity analysis are provided in S1 and S2 Tables in the supplementary material. Results differed only marginally from the analysis in the original sample.

## Discussion

Care for elderly lung cancer patients differs widely from care for patients under the age of 65 years, regardless of the presence of metastases at the time of lung cancer diagnosis. Elderly lung cancer patients receive fewer lung cancer-directed treatments than non-elderly patients. The proportion of patients not receiving any treatment in our study was around 40%, and even 66% among “old-old” patients with and without metastases, but only between 3% and 7% among “non-elderly” patients. An interesting finding was that the proportions of patients not receiving any tumor-directed treatments were higher in the group of patients without metastases. We hypothesized that a reason for this was a problem of misclassification of patients to this stratum. By refining our sample in the sensitivity analysis, we believe we were able to address this issue appropriately, as now proportions in the younger age groups were similar. In particular, the use of antineoplastic therapy declined with increasing age in both metastases strata. Recent studies and guidelines support the use of carboplatin-based doublets in fit elderly patients and single-agent treatment in less fit patients [14]. However, for patients aged 80 years and older, there are limited data from trials; therefore, specific recommendations for this age group are lacking [36]. The extreme drop seen between the “middle-old” and “old-old” might be related to the reluctance of patients and physicians to administer antineoplastic therapy in a setting with little evidence from trials.

Although rates of tumor resections in patients with metastases were comparable in all age groups, the share of patients receiving tumor resection dropped from 55% in the “non-elderly” to less than 12% in “old-old” patients. This drop was still apparent in the refined sample from the sensitivity analysis. As we adjusted for comorbidities, care level, and nursing home status, increasing frailty of older patients cannot fully justify this observation. In the early stages of lung cancer, tumor resection is also recommended in elderly patients [14] and should not be denied just because of calendar age. There is evidence that outcomes of tumor resection in lung cancer patients are similar irrespective of age, even in patients older than 80 years [13, 37].

The results of our study underline previous findings about less comprehensive tumor-directed treatment in elderly patients. Costa et al. found that the likelihood of patients ≥ 70 years of age receiving tumor resection, antineoplastic therapy, and radiotherapy was significantly lower than for younger patients [38]. Additionally, Palma et al. reported that age was a significant predictor of receiving tumor resection but not a significant factor influencing overall survival after this surgical procedure [39].

In addition to tumor-directed care, we found significant differences in the rate of palliative care in elderly lung cancer patients in Germany. Experts from the “European Organization for Research and Treatment of Cancer elderly task force” recommend that palliative care should be integrated into lung cancer therapy shortly after diagnosis in patients with advanced disease [14]. Also, Temel et al. show in their study that palliative care alongside usual oncologic care results in prolonged survival, improved health-related quality of life, and reduced depression [40]. However, in our sample, only between 20% and 35% of patients received any structured palliative care.

Further, we found that the likelihood of patients being treated with opioids declines significantly by age group. This might indicate insufficient pain management at more advanced age. A systematic review reports that pain in cancer is prevalent in 39–66% of patients depending on the stage of the disease [41]. It also suggests that experiencing pain from cancer or cancer treatment does not depend on age; therefore, differences in the prevalence of pain management should not occur. In contrast to our results, the review by Deandrea et al. found no consistent role of age and sex concerning low-level pain treatment in cancer patients [42]. However, other studies have shown that older patients are at a disadvantage when it comes to pain management [43].

Similar results were found in the treatment of depression. Studies report that female sex, severe illness, as well as poor functional and performance status predominantly predict depression in cancer patients [43–46]. Therefore, we would not expect significant age-related differences in the treatment of depression. Concerning treatment with antidepressants, Findley et al. report reduced treatment in elderly patients with cancer and depression [47], and Ashbury et al. found that cancer patients in general are insufficiently treated for depression [48].

Expenditures in our study refer to health insurance expenditures in the first 3 months after diagnosis and reflect the intensity of measures taken up right after diagnosis. Total expenditures were on average €6 000–14 000 with a significant decrease the higher the age group. This was true for almost all cost components. The biggest cost component was expenditures for hospitalizations. These results emphasize at the monetary level how the intensity of treatment decreases with advancing age.

A limitation of our study is that our data did not include information about the histology or stage of the tumor at diagnosis. Therefore, we cannot exclude that lower treatment intensity in elderly patients might result from more advanced stages in our age categories. However, a study from the UK found that patients in their 50s and 60s are more likely to be diagnosed with advanced lung cancer than older patients [49]. Furthermore, by stratifying our study sample by the presence of metastases and refining this definition in our sensitivity analysis, we believe we were able to approximate stage at diagnosis. All the above analyses showed stable results of differences in therapy with increasing age.

Another limitation is that we had no access to any measures of patient preferences. Previous research has shown that the desire for aggressive therapy is as high in octogenarians as in younger patients [36]. In addition, when patients did not receive guideline-recommended therapy, this was due to refusal in only 26% of cases, whereas 48% were not offered the therapy because of comorbidities or poor performance status, and 26% were not offered therapy because of their age [36]. Further, while patient preference may play a role in the use of tumor-directed therapy, it does not seem plausible that elderly patients prefer to receive less palliative care or pain relief. Previous research has found that elderly patients are at risk of undertreatment for pain, as their sensitivity to pain is underestimated, they are expected to be able to tolerate pain well, and there is a misconception about their ability to benefit from the use of opioids [43, 50]. Management of pain and depression are integral parts of palliative care and closely interlocked according to the concept of “total pain” in cancer by Cecily Saunders [51]. Therefore, a strength of our study is including palliative measures in addition to active tumor treatment. Furthermore, by also comparing expenditures in the phase directly following diagnosis, we reflect overall differences in the intensity of initial care. The outstanding feature of our analysis is the direct comparison of different age groups, with a classification algorithm established in gerontology. So far, studies have investigated differences in treatment given to elderly patients mostly by either including age as a continuous variable in a logistic regression or studying cohorts only including elderly patients [15, 52, 53]. Stratification for age has been done previously, but mostly specifying one large group as elderly patients, for example all patients > 70 years [28, 36]. Thus, non-linear effects of aging might not have been comprehensively addressed within previous work. Another strength of our study is the sample size of our dataset. It covers around 30% of German residents, and our study population includes patients from all 402 districts in Germany. The AOK SHI funds are part of the German SHI system, which covers around 86% of the population. Some 95% of all SHI services are defined by law and offered by all the distinct SHI funds. Therefore, our results are generalizable to at least the whole German population insured under SHI.

## Conclusion

In conclusion, our study describes a significant age gradient across all care aspects studied, affecting tumor-directed therapies as well as palliative care and the treatment of pain and depression. Evidence from this study suggests that this effect cannot be explained completely by patient preferences and a certain degree of undertreatment in elderly patients is plausible. As a majority of lung cancer patients are over the age of 65 years, this is of great public health concern. Although efforts to enhance palliative care in Germany have been made, lawmakers should further adjust public health policies to address these disparities.

## Supporting information

S1 Fig Adjusted odds ratio and incidence rate ratio of care among age groups of lung cancer patients diagnosed in 2009 in Germany. Days until first palliative care is reported as incidence rate ratio (IRR); all other outcomes are reported as odds ratios (OR). All IRR and ORs are adjusted for sex, nursing home residency, care level, Charlson comorbidity index, and rural vs. urban residence. CI = confidence interval, OR = odds ratio.
(TIFF)

S2 Fig Adjusted mean expenditures in the 3 months after diagnosis among age groups in lung cancer patients diagnosed in 2009 in Germany. All-cause and lung cancer-specific total, inpatient, outpatient, and medication expenditures within the 3 months after diagnosis reported as recycled predictions with 95% confidence intervals. Significance levels (* <0.05, ** <0.01, *** < 0.0001) indicate significant differences between the age groups “young-old” (65–74 years), “middle-old” (75–84 years), and “old-old” (≥ 85 years) and the reference group “non-elderly” (≤ 65 years). Lung cancer-specific expenditures relate to inpatient visits with a primary diagnosis of lung cancer, medications used in antineoplastic therapy or as supportive drugs (e.g., antiemetics, antianemics), and outpatient cases with a diagnosis of lung cancer.
(TIF)

S1 Table Unadjusted means and proportions of care among age groups of lung cancer patients with metastases and diagnosis confirmation, diagnosed in 2009 in Germany. Notes: Means and proportions of care in age groups “non-elderly”(≤ 65 years), “young-old” (65–74 years), “middle-old” (75–84 years), and “old-old” (≥ 85 years). P-values from Chi^2^ test for binary variables and Kruskal–Wallis test for continuous variables.
(DOCX)

S2 Table Adjusted odds ratio and incidence rate ratio of care among age groups of lung cancer patients with metastases and diagnosis confirmation, diagnosed in 2009 in Germany. Notes: Days until first palliative care are reported as incidence rate ratio (IRR); all other outcomes are reported as odds ratios (OR). All IRR and ORs are adjusted for sex, nursing home residency, care level, Charlson comorbidity index, and rural vs. urban residence. CI = confidence interval, OR = odds ratio.
(DOCX)
