# Supplementary figures and images for: “Age matters”—German claims data indicate disparities in lung cancer care between elderly and young patients
Source: PLoS One. 2019 Jun 12;14(6):e0217434. doi: 10.1371/journal.pone.0217434 (PMC6561547; doi:10.1371/journal.pone.0217434)

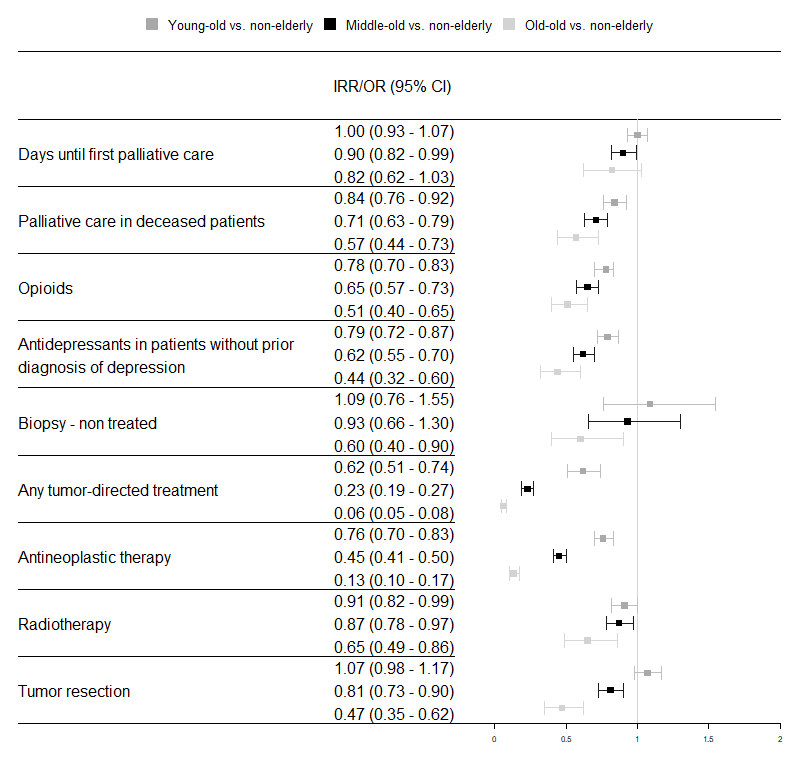

Supplement: S1 Fig — Days until first palliative care is reported as incidence rate ratio (IRR); all other outcomes are reported as odds ratios (OR). All IRR and ORs are adjusted for sex, nursing home residency, care level, Charlson comorbidity index, and rural vs. urban residence. CI = confidence interval, OR = odds ratio. (TIFF) [file pone.0217434.s001.tiff]

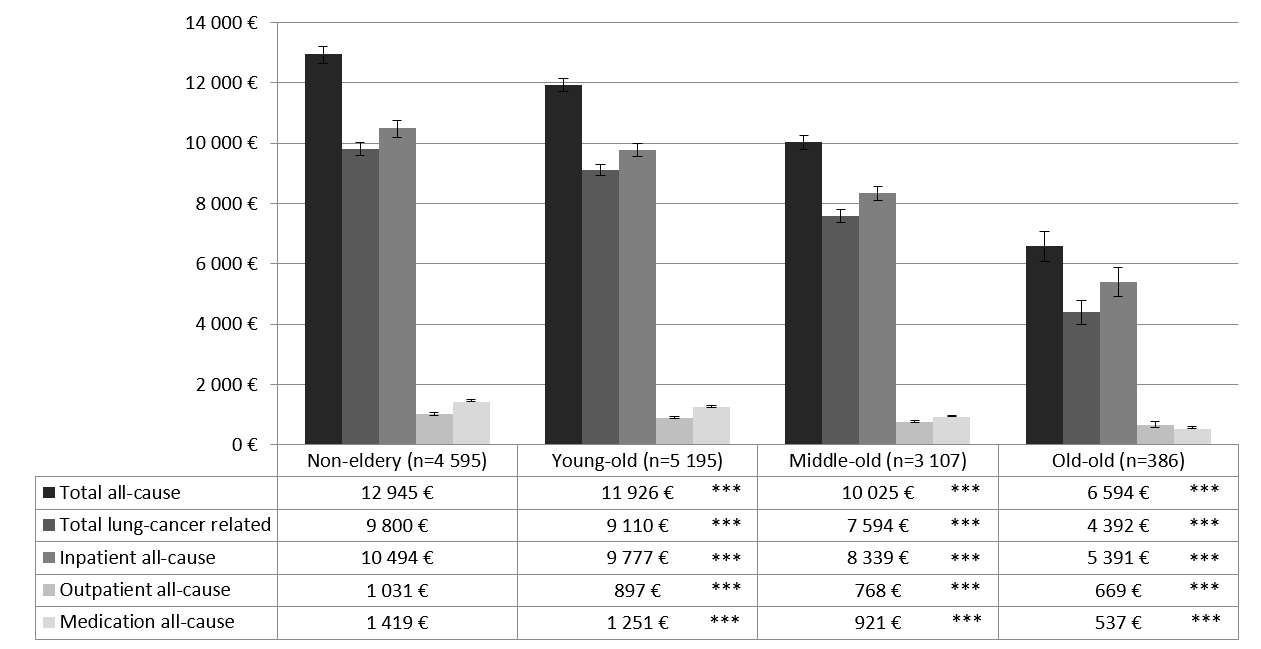

Supplement: S2 Fig — All-cause and lung cancer-specific total, inpatient, outpatient, and medication expenditures within the 3 months after diagnosis reported as recycled predictions with 95% confidence intervals. Significance levels (* <0.05, ** <0.01, *** < 0.0001) indicate significant differences between the age groups “young-old” (65–74 years), “middle-old” (75–84 years), and “old-old” (≥ 85 years) and the reference group “non-elderly” (≤ 65 years). Lung cancer-specific expenditures relate to inpatient visits with a primary diagnosis of lung cancer, medications used in antineoplastic therapy or as supportive drugs (e.g., antiemetics, antianemics), and outpatient cases with a diagnosis of lung cancer. (TIF) [file pone.0217434.s002.tif]
