# Supplementary material for: “Age matters”—German claims data indicate disparities in lung cancer care between elderly and young patients
Source: PLoS One. 2019 Jun 12;14(6):e0217434. doi: 10.1371/journal.pone.0217434 (PMC6561547; doi:10.1371/journal.pone.0217434)
Supplement: S1 Table — Notes: Means and proportions of care in age groups “non-elderly”(≤ 65 years), “young-old” (65–74 years), “middle-old” (75–84 years), and “old-old” (≥ 85 years). P-values from Chi2 test for binary variables and Kruskal–Wallis test for continuous variables. (DOCX) [file pone.0217434.s003.docx]

**S1 Table Unadjusted means and proportions of care among age groups of lung cancer patients with metastases and diagnosis confirmation, diagnosed in 2009 in Germany**

|  | Young (n=1 689) | Young-old (n=1 338) | Middle-old (n=1 157) | Old-old (n=142) | p-value |
| --- | --- | --- | --- | --- | --- |
| Structured palliative care in deceased patients in % (n) | 20.5 (274) | 20.5 (347) | 20.7 (239) | 23.9 (34) | 0.81 |
| of these mean time until structured palliative care (sd) | 475.4 (322) | 424.2 (320) | 380.7 (312) | 321.0 (245) | 0.001 |
| Opioid medication in % (n) | 74.7 (2 060) | 69.4 (2 009) | 68.1 (979) | 63.2 (84) | <0.0001 |
| Antidepressants in patients without prior diagnosis of depression in % (n) | 30.6 (774) | 26.0 (717) | 24.4 (344) | 17.3 (344) | <0.0001 |
| No tumor directed treatment in % (n) | 6.0 (80) | 9.0 (152) | 21.4 (248) | 59.9 (85) | <0.0001 |
| Antineoplastic therapy in % (n) | 47.5 (636) | 41.7 (705) | 32.8 (379) | 15.5 (22) | <0.0001 |
| Radiotherapy in % (n) | 14.3 (191) | 16.5 (279) | 20.7 (240) | 13.4 (19) | 0.0002 |
| Tumor resection in % (n) | 53.7 (719) | 50.6 (855) | 36.0 (416) | 14.1 (20) | <0.0001 |
