# Supplementary material for: “Age matters”—German claims data indicate disparities in lung cancer care between elderly and young patients
Source: PLoS One. 2019 Jun 12;14(6):e0217434. doi: 10.1371/journal.pone.0217434 (PMC6561547; doi:10.1371/journal.pone.0217434)
Supplement: S2 Table — Notes: Days until first palliative care are reported as incidence rate ratio (IRR); all other outcomes are reported as odds ratios (OR). All IRR and ORs are adjusted for sex, nursing home residency, care level, Charlson comorbidity index, and rural vs. urban residence. CI = confidence interval, OR = odds ratio. (DOCX) [file pone.0217434.s004.docx]

**S2 Table Adjusted odds ratio and incidence rate ratio of care among age groups of lung cancer patients with metastases and diagnosis confirmation, diagnosed in 2009 in Germany**

|  | Young-old vs. non-elderly | | | |  | Middle-old vs. non-elderly | | | |  | Old-old vs. non-elderly | | | |
| --- | --- | --- | --- | --- | --- | --- | --- | --- | --- | --- | --- | --- | --- | --- |
|  | OR/IRR | Lower CI | Upper CI | P-value |  | OR/IRR | Lower CI | Upper CI | P-value |  | OR/IRR | Lower CI | Upper CI | P-value |
| Structured palliative care in  deceased patients | 0.94 | 0.76 | 1.16 | 0.56 |  | 0.75 | 0.60 | 0.95 | **0.02** |  | 0.77 | 0.50 | 1.19 | 0.24 |
| of these mean time until  structured palliative care | 0.91 | 0.79 | 1.05 | 0.20 |  | 0.84 | 0.72 | 0.99 | **0.03** |  | 0.75 | 0.54 | 1.03 | 0.07 |
| Opioid medication | 0.80 | 0.69 | 0.94 | **0.005** |  | 0.77 | 0.65 | 0.92 | **0.003** |  | 0.54 | 0.37 | 0.77 | **0.001** |
| Antidepressants in patients  without prior diagnosis of depression | 0.74 | 0.62 | 0.88 | **0.001** |  | 0.50 | 0.40 | 0.61 | **<.0001** |  | 0.36 | 0.22 | 0.60 | **<.0001** |
| No tumor directed treatment | 0.68 | 0.51 | 0.91 | **0.01** |  | 0.29 | 0.22 | 0.39 | **<.0001** |  | 0.07 | 0.04 | 0.10 | **<.0001** |
| Antineoplastic therapy | 0.84 | 0.72 | 0.97 | **0.02** |  | 0.61 | 0.51 | 0.72 | **<.0001** |  | 0.26 | 0.16 | 0.41 | **<.0001** |
| Radiotherapy | 1.11 | 0.91 | 1.37 | 0.30 |  | 1.44 | 1.15 | 1.79 | **0.001** |  | 0.86 | 0.51 | 1.45 | 0.58 |
| Tumor resection | 0.92 | 0.79 | 1.07 | 0.28 |  | 0.56 | 0.47 | 0.66 | **<.0001** |  | 0.20 | 0.12 | 0.33 | **<.0001** |
